# Caller identification and characterization of individual humpback whale acoustic behaviour

**DOI:** 10.1098/rsos.231608

**Published:** 2024-03-13

**Authors:** Julia M. Zeh, Valeria Perez-Marrufo, Dana L. Adcock, Frants H. Jensen, Kaitlyn J. Knapp, Jooke Robbins, Jennifer E. Tackaberry, Mason Weinrich, Ari S. Friedlaender, David N. Wiley, Susan E. Parks

**Affiliations:** ^1^ Department of Biology, Syracuse University,107 College Place, Syracuse, NY 13244, USA; ^2^ Department of Ecoscience, Aarhus University, Frederiksborgvej 399, Roskilde, Denmark; ^3^ Biology Department, Woods Hole Oceanographic Institution, Woods Hole, MA 02543, USA; ^4^ Center for Coastal Studies, Provincetown, MA, USA; ^5^ Whale Center of New England, Gloucester, MA, USA; ^6^ Ocean Sciences & Institute of Marine Sciences, University of California Santa Cruz, Santa Cruz, CA, USA; ^7^ Stellwagen Bank National Marine Sanctuary, Scituate, MA, USA

**Keywords:** biologging, tags, call rate, caller ID, bouts, vocal exchange

## Abstract

Acoustic recording tags provide fine-scale data linking acoustic signalling with individual behaviour; however, when an animal is in a group, it is challenging to tease apart calls of conspecifics and identify which individuals produce each call. This, in turn, prohibits a robust assessment of individual acoustic behaviour including call rates and silent periods, call bout production within and between individuals, and caller location. To overcome this challenge, we simultaneously instrumented small groups of humpback whales on a western North Atlantic feeding ground with sound and movement recording tags. This approach enabled a comparison of the relative amplitude of each call across individuals to infer caller identity for 97% of calls. We recorded variable call rates across individuals (mean = 23 calls/h) and groups (mean = 55 calls/h). Calls were produced throughout dives, and most calls were produced in bouts with short inter-call intervals of 2.2 s. Most calls received a likely response from a conspecific within 100 s. This caller identification (ID) method facilitates studying both individual- and group-level acoustic behaviour, yielding novel results about the nature of sequence production and vocal exchanges in humpback whale social calls. Future studies can expand on these caller ID methods for understanding intra-group communication across taxa.

## 1. Introduction

In studies of animal communication, it is valuable to be able to differentiate the sender and receiver of a given signal [1]. Once caller identity has been assigned, more detailed information about the vocal behaviour of a species can be inferred, including individual call rates, the timing of signal production, and the production of acoustic sequences within and between individuals. However, in naturalistic social settings across taxa in both the lab and in the field, assigning acoustic signals to the individual that produced them can be challenging [2,3]. Unless an animal gives an obvious visual cue when vocalizing, caller identification (ID) requires either highly precise sound source localization (e.g. [2,4]) or some other method of differentiating the calls of one individual from those of conspecifics in the vicinity. Animal-borne tags containing movement and acoustic sensors provide valuable fine-scale data to link individual sound production and behaviour [5]. However, these acoustic sensors record all detectable sounds from both the tagged animal and nearby conspecifics [5]. In social groups, conspecifics are often in close proximity to the tagged animal; therefore, calls from other animals present challenges for caller ID. This is especially problematic for studies of social animals and underwater sound production, since sound propagates efficiently and rapidly through water, resulting in a high probability of detecting nearby vocalizing conspecifics.

While past studies have used various methods for caller ID, most of these methods remain problematic or are limited to only certain taxa. For example, the angle of arrival of recorded sounds on stereo hydrophones in tags has been used for caller ID [6–9], sometimes in concert with vertical separations from the social group [10,11]. While calculations of the angle of arrival of sounds have been useful for assigning calls as focal (i.e. from the tagged animal) or non-focal for the high-frequency clicks and whistles of odontocete species [6–9], these methods prove problematic for low-frequency baleen whale calls, whose longer wavelengths and gradual amplitude onset hinder localization with narrow inter-hydrophone spacing.

The use of individual identity information in recorded sounds can also be used as caller ID for some species. For example, individual spectral features in goat (*Capra aegagrus hircus*) vocalizations have allowed for caller ID [12], as has the inter-pulse interval in sperm whale (*Physeter macrocephalus*) codas [13,14]. These methods are only possible in select situations when animal vocalizations contain individual identity cues, and these cues are known. No such methods currently exist for robust individual identification from baleen whale calls.

In contrast, signal-to-noise ratio (SNR) thresholds have been frequently used in assigning caller ID in baleen whale tag data (e.g. [15,16]), but this method can be problematic given that individuals vary the source level of their sounds; quiet sounds may come from the tagged animal, and nearby conspecifics may produce calls detected on the tag with high SNR [3]. SNR measurements will also depend on tag attachment location as well as the call type and background masking noise.

Finally, some studies have used signatures of very low-frequency sounds picked up by the tag accelerometer data for caller ID [3,17–19]. While accelerometer signatures of calling behaviour have shown promise, these methods can still be ambiguous since accelerometers have been shown to pick up calls from both the tagged whale and the nearby conspecifics [18]. Furthermore, sufficiently high-resolution accelerometer data would be necessary to detect higher frequency baleen whale calls, and even then, the mechanism involved in accelerometer detection of vocalizations is still unclear and not all focal calls register on the accelerometer [3].

More recently, Kragh *et al.* [7] distinguished bottlenose dolphin (*Tursiops truncatus*) whistles produced by the tagged individual from those produced by non-focal animals via a combination of the angle of arrival of whistles and, when simultaneously tagged pairs were tightly associated, differences in call intensity were recorded across the two tags. Comparisons of call amplitude across tags require tags deployed on all individuals in a social group, but show promise for studies of baleen whale calls. Here, we show how this method can be used to distinguish focal and non-focal calls in tag data from humpback whales (*Megaptera novaeangliae*).

Humpback whales are found across the globe and migrate annually between low-latitude breeding grounds and high-latitude feeding grounds [20]. They are acoustically active throughout their range, producing a variety of social sounds across various contexts, including in large aggregations (e.g. [21,22]). On their feeding grounds, humpback whales can be found in large aggregations and are vocally active across different contexts [22]. Males also produce a complex, hierarchically structured song, which is recorded most often on the breeding grounds [23]. The song is produced in rhythmically repeating sequences, which can facilitate tracking individual singers and teasing apart individual songs (e.g. [24]). In addition to songs, there is ample evidence of social calls produced in bouts by individuals (e.g. [25]). In the South Pacific, migrating humpback whales were shown to produce most of their social calls in bouts with 3.9 s or less between calls, based on an SNR threshold for estimating which calls were focal [25,26]. Bouts are widely variable in duration, context and call types, but there is some evidence of syntactical rules governing the order of call types in a bout [25].

Humpback whales are challenging subjects for caller ID because, in addition to being baleen whales with far-reaching low-frequency calls, they are often vocally active in social settings when many individuals are vocalizing near one another. Thus, little data exist that have allowed for quantitative analysis of the nature of individual bout production or vocal exchanges. In addition to call bouts from a single individual, inter-individual call bouts are involved in vocal exchanges. While some animals exhibit simple call and response dynamics, others have shown evidence of temporal rules in call exchanges indicative of turn-taking and temporal coordination (e.g. [27,28]). These turn-taking rules involve limited or no interruptions and describe the periodicity of vocal exchanges, in line with a similar analysis of coordination in human conversation [28]. Group vocal coordination may also arise from individual rules related to call inhibition and excitation in response to conspecific vocalizations [27]. In part due to challenges with caller ID, quantitative descriptions of vocal exchanges, also sometimes referred to as counter calling, are lacking for humpback whales.

Without robust caller ID methods, it is difficult to study individual vocal behaviour and calculate individual call rates. Call rates are increasingly important for passive acoustic monitoring (PAM) and acoustic density estimation (e.g. [29]), especially in the context of vocal exchanges. The behavioural context of signal production on an individual level, such as the depth at which animals are vocalizing, is similarly challenging to describe but important for modelling signal detection range to use with PAM and density estimation.

In this study, we test whether we can use calls’ received levels (RLs) from acoustic recording tags simultaneously deployed on all animals in a social group to assign caller identity. We then describe individual vocal behaviour and explore vocal exchanges in groups (pairs and trios) of North Atlantic humpback whales on the Gulf of Maine feeding ground. Specifically, we look at how individual vocal behaviour relates to individual movement behaviour by calculating the depth at which individuals vocalize. Furthermore, we investigate the acoustic context of individual calls by testing for and characterizing bout production and call timing in vocal exchanges, all of which could not be assessed without robust caller ID methods.

## 2. Materials and methods

### 2.1. Data collection

Sound and movement data were collected from humpback whales in the Gulf of Maine in and around Stellwagen Bank National Marine Sanctuary in the Western North Atlantic between 41.5 and 43.2°N and 69.3 and 70.5°W. Archival digital acoustic recording tags (Dtag version 2 [30]) were attached via suction cups from a handheld 7–15 m pole in July 2006–2009 [31]. Dtag hydrophones recorded at a sampling rate of either 64 or 96 kHz and orientation sensors recorded at a sampling rate of 50 Hz, which were decimated to 5 Hz for analysis. We did not examine the accelerometer data for signatures of vocalizations because the sampling rate of the accelerometers used in this study was too low; a 50 Hz sampling rate would only allow detection of sounds up to 25 Hz, and most humpback whale vocalizations are >100 Hz [22]. Behavioural observations, including social affiliations, were also collected concurrently from a small inflatable vessel at a distance of a few hundred metres away (e.g. 32, 33). A handheld GPS onboard the vessel was used to record the location of tag deployments. Individual whales were identified based on the unique shape and pigmentation pattern of their ventral flukes [34]. They were photographed and matched to photo-identification catalogues from long-term studies led by the Center for Coastal Studies and the former Whale Center of New England. Whales were classified as male or female based on molecular sex determination [35,36], a photograph of the genital slit, or, in the case of females, a calving history [37]. Calves were classified based on their size, stereotypical behaviours and close consistent association with a mature female (the mother). The age class of other individuals was assigned from longitudinal data on the exact or minimum age of each individual. With the exception of the calves, all the individuals in the study were at least 5 years old and, therefore, considered adults [38–40].

### 2.2. Acoustic analysis

#### 2.2.1. Focal call assignment

To ensure that we could accurately assign calls to specific individuals in the group, we only used tag data from periods of time when (1) all whales in a group were equipped with tags, (2) no untagged whales were associated with or in close proximity to the group (<500 m), and (3) visual observers were recording behavioural focal follow data to confirm the social associations and behavioural context of the tagged whales. Most data analysis began at the time point when the last tag in the group was deployed. The analysis ended when behavioural observations stopped, another whale joined the group or a tag detached from one of the whales in the group. During these analysis periods, we manually detected all humpback whale vocalizations and compared the relative RL of the signal across all the tags in the group to identify which animal was calling. A call should have the greatest RL on the tag attached to the whale producing the sound, regardless of the sound source level, because that tag would be closest to the sound source.

**Figure 1.**
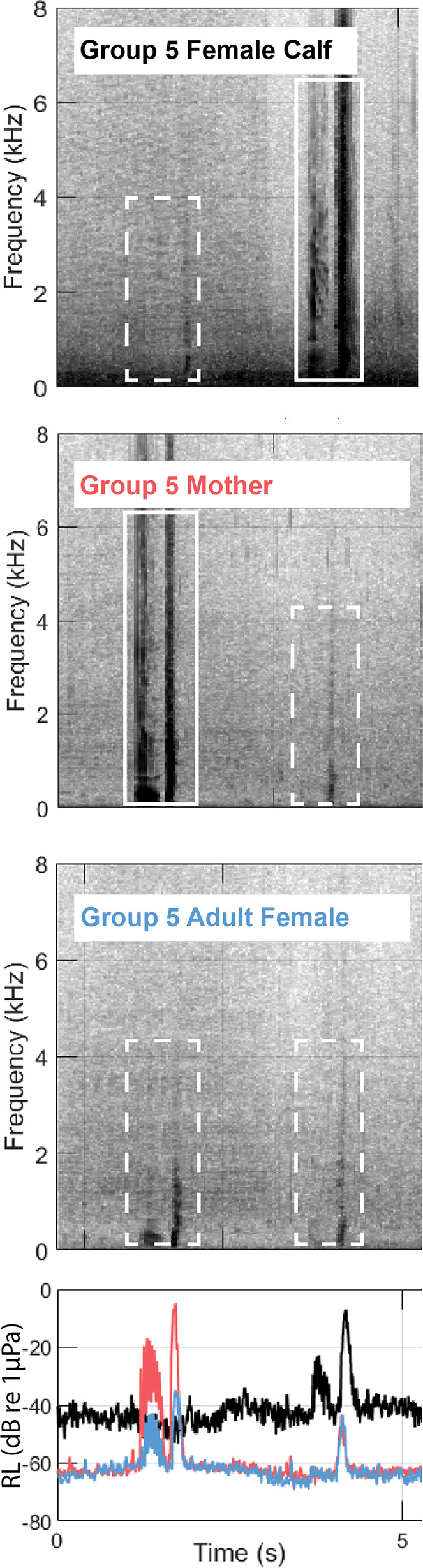
Spectrograms and received level (RL) plot showing two vocalizations recorded on all three tags in Group 5. Dashed boxes show the non-focal instances of the calls, and solid boxes show the focal instances of each call. The colours of the text labels on the spectrogram correspond to the colours of the lines in the RL plot.

Experienced analysts manually selected individual humpback whale calls in the acoustic record of each tag. Tag acoustic records were analysed both individually using Raven Pro v2.0 [41] and simultaneously in MATLAB 2019b [42] using custom scripts [43] modified from the Dtag toolbox (animaltags.org). All humpback whale calls were selected in Raven Pro, regardless of whether the analyst thought the calls were from the tagged individual. Single and simultaneous tag audits were conducted by separate analysts, and analysts were blind to the results of the analysis with the other method. All sound files were thus browsed by at least two experienced analysts to reduce false positives and false negatives. Once detections from the two analysts were combined, simultaneous tag analysis was used to identify focal (i.e. originating from the tagged whale) and non-focal (i.e. originating from a whale other than the tagged whale) calls in the tag record based on relative call intensity across tags [7]. This involved plotting spectrograms and relative intensity plots from time-aligned acoustic data from all concurrent tags [43]. For each manually selected call, the spectrogram(s) of the other tag(s) were examined for instances of the same call (figure 1). If calls were not recorded on the other tag(s) in the group, they were assumed to be focal calls. If calls were recorded on the other tag(s) in the group, relative intensity was compared and calls were assigned as either focal (when relative intensity was highest on that tag), non-focal (when relative intensity was lower than it was on another tag) or indeterminate (when there was no clear difference in relative intensity across tags). When one tag was obscured due to noise, including surfacing noise, the call was marked focal for the tag where it was visible on the spectrogram. Indeterminate calls may have been produced by a tagged whale when in very close proximity to another tagged whale or may have been produced by a whale outside the group and recorded with the same intensity on all tags. We also noted whether calls were detected on multiple tags and whether noise (e.g. flow noise and splashing noise during surfacing) was present on one of the tags that significantly affected the SNR of a call.

We measured the RL of focal and non-focal calls in MATLAB by first decimating the audio to 12 kHz and then applying a 500 Hz high-pass filter to reduce flow noise. We only measured RL for those focal and non-focal calls that did not overlap temporally with other sources of noise or with other social calls. For those calls where we could measure the signal, we measured the root-mean-squared (rms) RL using the *rms* function in MATLAB based on a 90% energy window. We then converted this value to dB re 1 µPa using a nominal hydrophone sensitivity of −171 dB re 1 V/µPa [22]. After making RL measurements, we paired up focal and non-focal instances of the same call to measure the difference in RL of the same call when it was recorded across multiple tags; however, it is important to note that call RLs also depend on tag placement on an animal, and variation in tag placement across deployments would thus affect these calculated differences. All statistical analyses were done in R version 4.1.2 [44].

Only calls labelled focal were retained for further analysis, and we used these data as well as the analysis duration to calculate raw call rates at both the individual and group levels. We also calculated the proportion of the total analysis period that was silent (i.e. contained no call detections from any individual in the group).

#### 2.2.2. Vocal exchanges and bout analysis

To understand the communicative context of calling behaviour, we investigated call timing both within and between individuals by looking at individual call bouts and inter-individual vocal exchanges. We conducted a bout analysis by calculating a bout end criterion (BEC), which determines a threshold for defining calls as part of a bout [45]. First, we calculated inter-call intervals (ICIs) from the start of one call to the start of the next call from the same individual. We then log-transformed the ICI data and used the R package *diveMove* to determine the BEC using the maximum likelihood estimation method [46,47]. The package *diveMove* was developed to look at dive bouts using dive intervals, but the methods are applicable for intervals and bouts of any behavioural parameters. The BEC method assumes that the distribution of behavioural data combines two or more Poisson processes, including fast processes (calls within a bout) and slow processes (calls in separate bouts). The BEC is calculated as the point where the distribution switches between these two processes and has been described as a ‘broken-stick’ model [45]. After calculating the BEC, we classified calls with ICIs less than the BEC as bouts.

We examined vocal exchanges in groups by looking at relative call timing between individuals. We calculated the between-individual ICI as the difference between the start time of a call and the start time of the next call made by a different individual. We then used these inter-individual ICI data to calculate a probability density function and integrated over the function to get the area under the curve (AUC).

#### 2.2.3. Movement analysis

Accelerometer, magnetometer and pressure sensor data were calibrated and processed using custom-written MATLAB scripts (animaltags.org). The depth of call production was also calculated for all focal calls across all individuals by comparing the time of call production to the pressure time series from the tag. Maximum dive depths were calculated for each dive and each individual to investigate the call production depth relative to the dive depth. To assess dive and call production depths relative to bathymetry, we also report the estimated seafloor depth based on GPS coordinates from where the tag was deployed on the whale.

## 3. Results

In total, we analysed 46 h 52 min of tag data for which we had synchronous tags on all whales in a given group with concurrent behavioural observations, which allowed for RL comparisons and caller ID. This included 16 tags from 7 distinct groups of whales, with 12 females and 4 males. These 16 whales also included 3 calves and 3 mothers. Most of the whales were foraging for most of the tag duration, although some were also travelling or resting.

### 3.1. Focal call assignment

**Figure 2.**
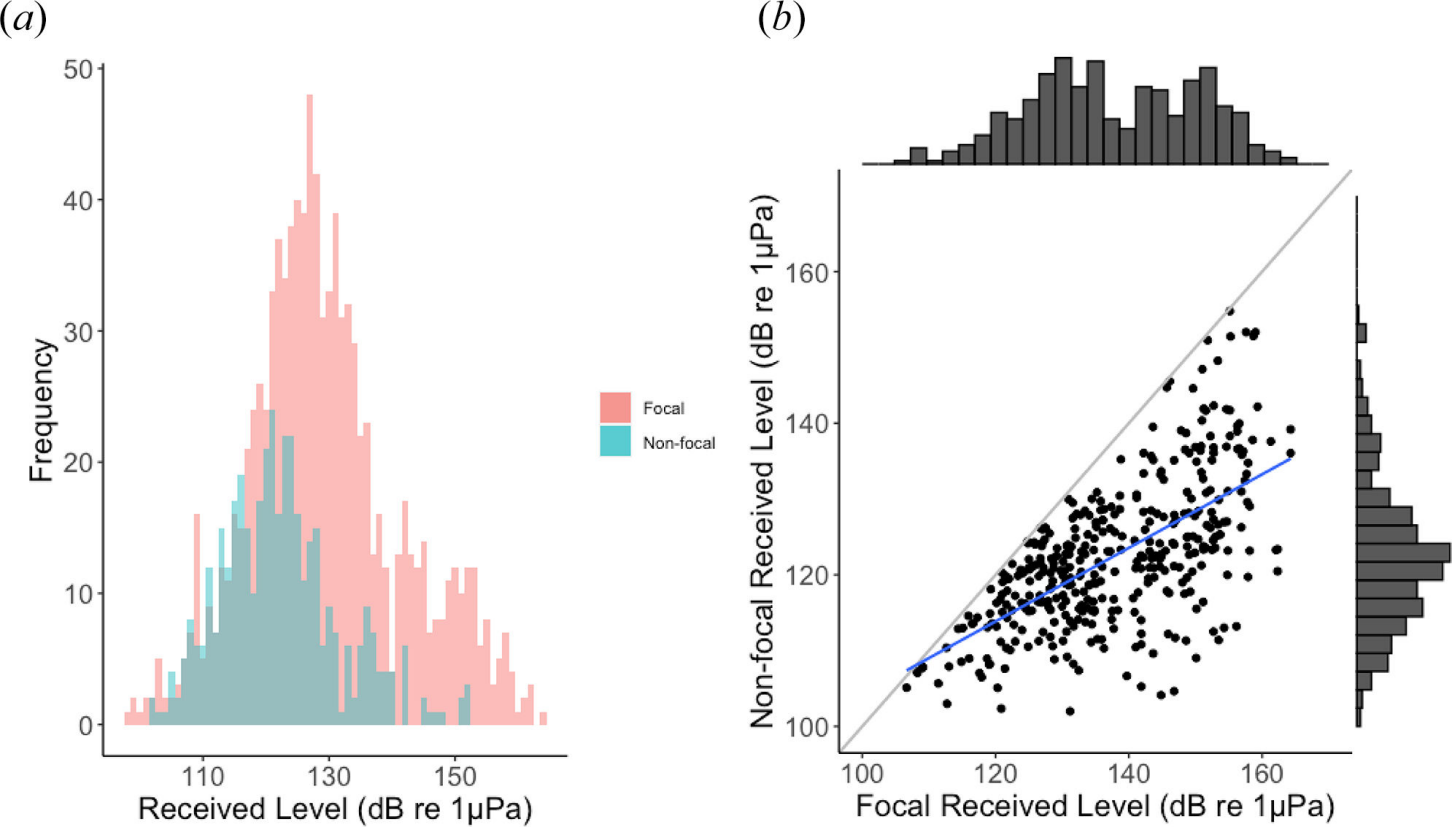
RLs of focal and non-focal calls. (*a*) Histogram showing the distribution of RLs of focal (red) and non-focal (teal) calls overlaid on the same plot. (*b*) Scatterplot for calls that were recorded across multiple tags, and the non-focal RL is plotted against the corresponding focal RL of the same call. The identity line is shown in grey, and a linear regression line for the data is shown in blue. Marginal histograms show the distribution of focal RLs (x-axis) and non-focal RLs (y-axis).

We detected 1035 total unique calls across all 16 tags and were able to use RL comparisons across tags (i.e. figure 1) to assign 1008 (97%) of those calls to an individual (table 1). Some individuals did not produce any calls, while others called over 300 times (table 1). There were 27 calls (3%) that could not be assigned to an individual because of the similarity in RL across tags. We also identified 490 non-focal calls in total, which were the quietest instances of a call when it was detectable on multiple tag records. Of the 1035 total unique calls detected across all individuals, 414 calls (40%) were detected across multiple tags and 621 calls (60%) were only detected on one tag. The average RL of all measured focal calls was 129 dB re 1 µPa and the average RL of all non-focal calls was 122 dB re 1 µPa. The mean difference in the RL of a call recorded across multiple tags was 15 dB. The distribution of RLs of non-focal calls overlaps entirely with the distribution of RLs of focal calls (figure 2). We did not notice any trends in RL relative to whether the calls were detected on multiple tags or not.

**Table 1.** Summary of tag data, class of individuals tagged, analysis duration and total focal calls detected. Totals represent individual detections and are not the same as unique sounds; there is an overlap in calls that are counted as focal on one tag and non-focal on another or as indeterminate on multiple tags. For example, although there were 58 detections of indeterminate calls, this represents only 27 unique calls that could not be attributed to a specific individual.

**date**	**group**	**analysis duration (hh:mm)**	**whale class**	**total number of focal calls**	**total number of non-focal calls**	**total number of indeterminate calls**	**focal call rate (calls/h)**
19 Jul 2006	1	1:28	adult female 1	0	15	0	0
			adult female 2	20	0	0	13.6
17 Jul 2007	2	2:40	male calf	3	0	0	1.1
			mother	0	1	0	0
7 Jul 2008	3	2:27	adult male	8	5	0	3.3
			adult female	12	8	0	4.9
14 Jul 2008	4	0:31	adult female	46	9	1	89
			adult male	15	15	1	29
22 Jul 2009	5	3:47	female calf	15	28	11	2.2
			mother	18	37	11	2.6
			adult female	77	11	8	11.1
20 Jul 2009	6	6:55	adult female	335	173	10	88.5
			female calf	314	75	12	83
			mother	139	113	4	36.7
29 Jul 2009	7	0:17	adult female	0	0	0	0
			adult male	6	0	0	21.2

Additionally, 489 of the detected focal calls (47%) occurred on one tag when noise was present on the other tag(s), so it is possible that call detection on multiple tags was prevented due to masking. However, the amplitude of the noise on the other tag(s) in these cases was generally low and would likely have only masked non-focal calls or some low-amplitude focal calls, reducing the risk of this type of error.

The hourly call rate, based on the analysis duration and number of focal calls detected, ranged from 0 to 87 calls per hour (table 1). The average call rate across individuals was 23 calls per hour and across groups was 55 calls per hour. On average across tags, 71% of the analysis period was silent and contained no call detections. The longest periods of silence across tags ranged from 278 s to 3.62 h.

### 3.2. Bout analysis

**Figure 3.**
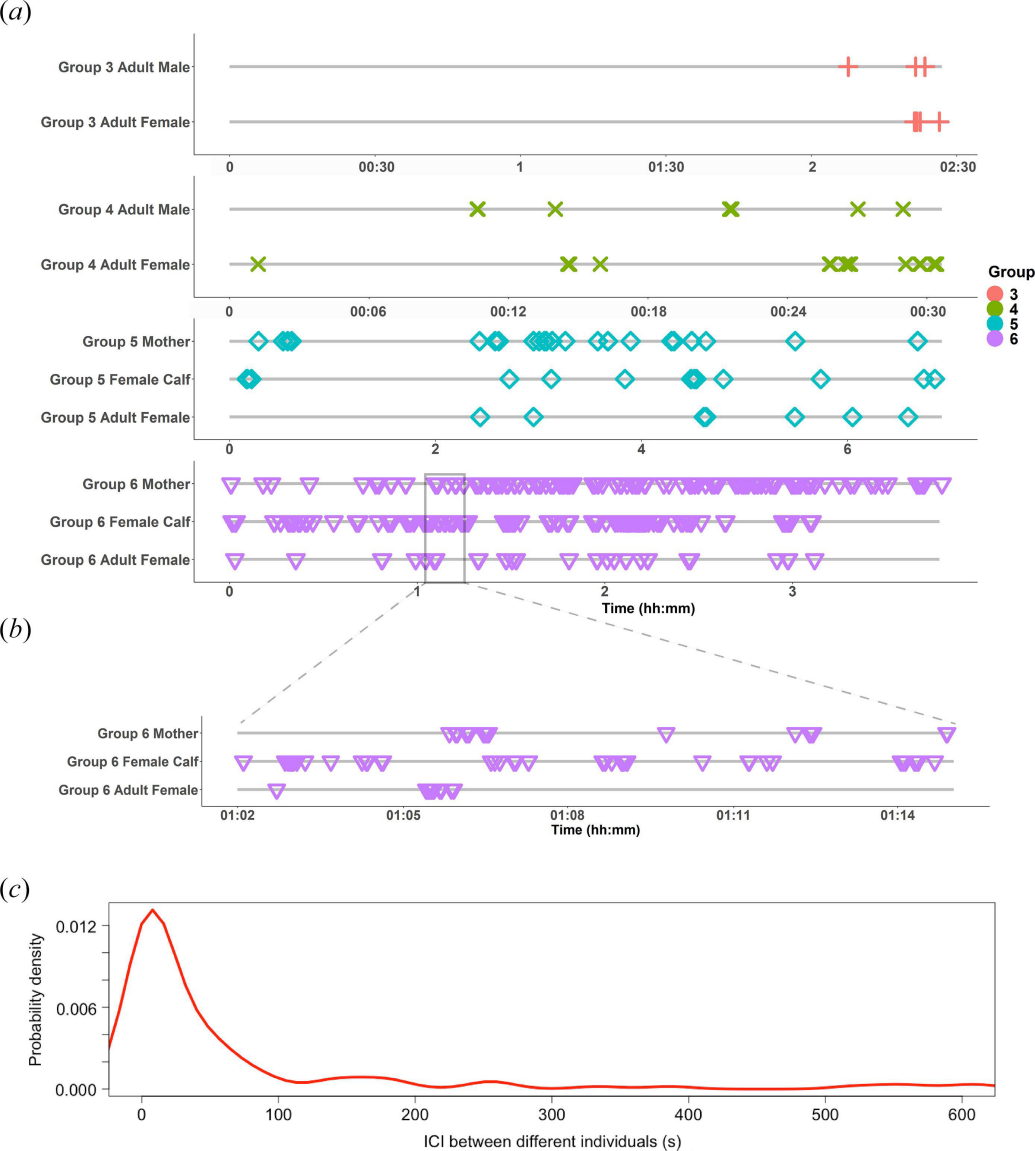
Relative timing of focal call production across individuals. (*a*) Timelines of focal call occurrences (coloured symbols) on each tag relative to the analysis period (grey line). Colours and symbols correspond to each different group. Groups 1, 2 and 7 are not shown because only one of the animals in the group vocalized. (*b*) Zoomed-in portion of a period (01:02 to 01:15) from Group 6, showing call occurrence timing. (*c*) Probability density curve of the ICI between different individuals. The AUC from 0 to 100 s is 0.58.

The BEC for this dataset is 2.2 s, meaning that any calls with an ICI of less than 2.2 s were classified as part of bouts, while those with greater ICIs were not. On average, across individuals, 79% (±15% s.d.) of calls were produced as part of bouts. Bouts were made up of 2–6 calls on average, and individuals produced between 0 and 69 total bouts (table 2). Bout rates ranged from about 0 to 14 bouts per hour (table 2). Inter-individual ICIs ranged from 0.05 to about 8000 s. The AUC between 0 and 100 s for the probability density function was 0.58, meaning that 58% of the time, a call from one whale was followed by a call from a different whale within 100 s (figure 3).

**Table 2.** Number of bouts, bout rate and mean number of calls per bout for all tags.

**group**	**analysis duration (hh:mm)**	**whale class**	**total number of bouts**	**bout rate (bouts per hour)**	**mean number of calls per bout**
1	1:28	adult female 1	0	0	0
		adult female 2	3	2	6
2	2:40	male calf	1	0.4	2
		mother	0	0	0
3	2:27	adult male	2	0.8	3.5
		adult female	3	1.2	3.7
4	0:31	adult female	7	13.5	6
		adult male	2	3.9	3.5
5	3:47	female calf	4	1	2.8
		mother	4	1	2
		adult female	17	4.5	3.9
6	6:55	adult female	69	6.6	3
		female calf	40	3.1	5.3
		mother	19	3.5	6.3
7	0:17	adult female	0	0	0
		adult male	1	11.3	3

### 3.3. Movement analysis

**Figure 4.**
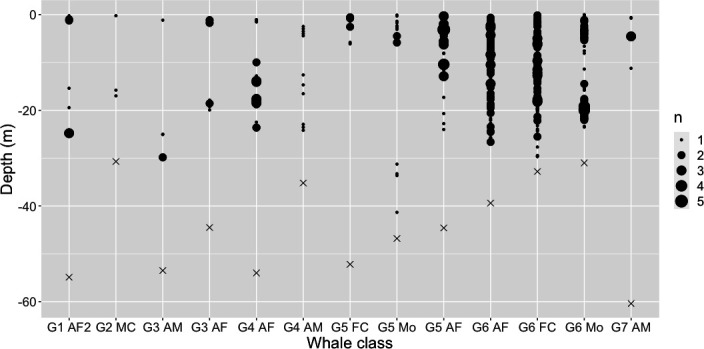
Depth of call production for all focal calls for each individual. Point size represents the number of calls at that depth, and × marks maximum dive depth of that individual. Whale class is abbreviated to group (G) and number, plus two letters to mark sex, female reproductive status, and age class. M and F are used to denote male and female, A and C are used to denote adult and calf and Mo denotes a mother. A number was added at the end when needed.

Tagged whales vocalized across the full range of dive depths observed on the tags (figure 4). Thirteen per cent of all calls were produced at/near the surface (i.e. less than 2 m depth) and the rest were produced at various points during dives. The maximum depth of call production was 41 m, the minimum depth of call production was at the surface, and the mean depth of call production was 11 m (±7 m s.d.). Maximum dive depths ranged between 30 and 60 m and the mean maximum dive depth across individuals was 45 m. The average water depth at the location of the tag deployments was approximately 62 m across tags (minimum water depth: 33 m, maximum: 125 m). There were no observed differences in the depth of call production between calves and adults, although all groups with calves were tagged in water depths of 30–40 m, while adult-only groups were tagged in water depths of 60–125 m.

## 4. Discussion

Using this approach of comparing relative RLs of calls recorded across tags on all whales in a group, we successfully assigned calls to callers for approximately 97% of calls in the dataset. Both focal and non-focal calls were recorded over a wide range of RLs, and the low end of the focal RL range was lower than that of the non-focal RL range. This indicates that although simultaneous tags often show a clear difference in call RLs across tags, and even though the distribution of non-focal RLs overlaps mostly with the lower end of the distribution of focal RLs, focal and non-focal calls occupy similar RL levels within a single tag. This is likely because whales vocalize at varying source levels both within and across call types, as has been described for humpback whale song [3]. There is still a level of uncertainty with RL measurements, as there are differences in the tag location that may impact tag differences in RL, and other propagation effects may cause RL to vary depending on the environment. Additionally, the relative proximity of the whales to each other and their respective tags can influence call RLs, meaning that tag and whale locations have the potential to confound caller ID based solely on relative call intensity. Thus, the range of RLs shown here is meant to be representative but could still reflect these measurement uncertainties. The range of RL results for focal and non-focal tags provides additional evidence that while an SNR threshold for determining focal calls may work in some cases, it may not always be robust enough to distinguish between focal and non-focal calls. This is probably true for other taxa as well, since it is common for animal vocalizations to vary in amplitude across individuals and across contexts within an individual [48].

After assigning focal calls based on the relative RL, we were able to calculate the call rate at both the individual and group levels. Since some calls were classified as indeterminate, actual call rates may be higher than our estimates, but this would primarily impact those tags with already high call rates. The call rate varied widely across individuals, with a mean individual call rate of about 23 calls per hour, but with some tag records that did not contain any vocalizations and others that contained over 80 calls per hour. Similarly, the group-level call rate varied, with a mean group call rate of 55 calls per hour, but with some group call rates as low as 1 call per hour and some group call rates as high as 208 calls per hour. Since humpback whales also seem to produce most of their calls in bouts (79% of calls produced in bouts), the call rate is not evenly distributed across recording time. It may be useful for future studies to report additional statistics such as the bout rate and average bout length to better represent the call rate over time. The call rate and bout rate have important implications for PAM, particularly for passive acoustic density estimation [29]. We also found temporal associations in calling activity, meaning that most of the time, when one individual vocalizes, another individual vocalizes shortly after. Future research can investigate whether this is representative of coordinated call and response behaviour. This behaviour also implies that the call rate is likely dependent on the social context, which is also important to consider in interpreting passive acoustic data, especially for density estimation.

Our bout production results are in alignment with previous results from this species on migration in Australia [25,26]. Growing evidence of bout production by humpback whales across populations and habitats suggests that more research should investigate the social and behavioural context of these bouts. Additional data will allow for the development of functional hypotheses as well as an understanding of bout characteristics like syntax and rhythm and how these aspects of social call bouts compare to humpback whale songs. In other species, acoustic sequences have been found to contain information related to signaller identity or context (e.g. [49,50]). Understanding the content and function of acoustic sequences is a growing area of research in animal behaviour, and development of analytical techniques for answering questions related to acoustic sequences is ongoing [51]. We found a BEC of 2.2 s, which, along with the previously calculated BEC of 3.9 s from the South Pacific [25], means that humpback whales are producing bouts with short ICIs. ICIs in vocal bouts may encode additional information and, in some cases, may be indicative of social situations and arousal [52, 53]. Humpback whale songs exhibit variable inter-unit intervals that on average range from about 0.5 to 2.5 s [53,54]. Thus, silent durations between sounds are similar in humpback whale social call bouts and songs, although song inter-unit intervals may tend to be shorter. In contrast, the inter-unit intervals in blue whale songs are between about 5 and 14 s on average [55].

Since vocal exchanges are challenging to study without caller ID, this study is novel in our investigation of the timing of vocal production between individuals in this species. We found evidence that humpback whales are regularly calling back and forth with inter-individual call intervals of 100 s or less. Timing in vocal exchanges can indicate cooperative and turn-taking dynamics and mechanisms [27,28] or can encode information like dominance or internal state [52,56]. In common marmosets (*Callithrix jacchus*), the measured median time interval in vocal exchanges is about 5 s, which matches coupled oscillator dynamic predictions [28]. Future research can investigate the dynamics of humpback whale vocal exchanges in more depth and test hypotheses related to the information contained in call timing (e.g. in mother-calf contact calling and group cohesion) as well as the mechanisms underlying call timing, like coupled oscillator dynamics or other models as demonstrated in other taxa, including common marmosets [28], meerkats (*Suricata suricatta* [27]) and humans.

An additional factor that is important for PAM is the depth at which marine animals are calling. For example, right whales predominantly signal near the surface [16] and blue whales have been found to predominantly call at shallow depths (<30 m), even while making deep dives (>100 m [15]). Alternatively, short-finned pilot whales vocalize both while socializing at the surface and during deep (up to 800 m) foraging dives [10]. Here, we found that humpback whales are calling at various depths throughout their dives in this shallow habitat. For humpback whales, evidence of call production throughout the water column may indicate the use of vocalizations across different behavioural contexts (i.e. coordinated foraging and social interaction) across depths, and future research could further investigate behavioural context and function of different call types relative to the location in the water column. This is useful for understanding the risk of anthropogenic disturbances like entanglement or ship strikes, as well as for modelling acoustic propagation and detection range of vocalizations for acoustic monitoring.

Simultaneously equipping all the individuals in a social group with recorders has the potential to be useful across taxa for studies of individual and group-level acoustic behaviour and facilitates the study of social interactions. For humpback whales, this method can provide fine-scale communication data, which will enable studies that were not previously possible, including investigations of variation in call rates, call exchanges, and ICIs within and between age classes, sexes, behavioural states and social contexts. Where possible, future studies requiring robust caller ID can prioritize deploying tags on all the animals in a group to compare call RLs across recorders. However, future research on additional methods for differentiating individual callers in acoustic data remains important. Deploying acoustic recorders on all individuals in a group can be restrictive, especially when social context changes frequently or when group size exceeds the number of tags available for deployment. An additional requirement of simultaneous tagging for caller ID is concurrent behavioural observations to track social affiliations.

This study provides evidence of the feasibility of using simultaneous tag data for caller ID with small groups of baleen whales and offers a more robust method for identifying focal calls than an SNR threshold. It will also be useful for future studies to pair this simultaneous tag method with the analysis of accelerometer records for signatures of vocalizations (as in [3,17,18]) and thus cross-validate different methods for identifying calls from tagged baleen whales. Using this method, we were able to gain insight into individual humpback whale acoustic behaviour, including a description of ICIs between and within individuals, which provides preliminary baseline data that can be used for future research related to rhythm, sequence production, and cooperative behaviour. These data also allowed for the calculation of call rates and call production as it relates to dive behaviour, which will be useful for conservation applications including PAM and density estimation.

## Data Availability

Data are available online at Dryad Digital Repository [57].

## References

[1] Demartsev V , Gersick AS , Jensen FH , Thomas M , Roch MA , Manser MB , Strandburg‐Peshkin A . 2023 Signalling in groups: new tools for the integration of animal communication and collective movement. Methods Ecol. Evol. **14**, 1852–1863. (10.1111/2041-210X.13939)

[2] Heckman JJ , Proville R , Heckman GJ , Azarfar A , Celikel T , Englitz B . 2017 High-precision spatial localization of mouse vocalizations during social interaction. Sci. Rep. **7**, 3017. (10.1038/s41598-017-02954-z) 28592832 PMC5462771

[3] Stimpert AK , Lammers MO , Pack AA , Au WWL . 2020 Variations in received levels on a sound and movement tag on a singing humpback whale: implications for caller identification. J. Acoust. Soc. Am. **147**, 3684–3690. (10.1121/10.0001306) 32486778

[4] Miller PJO , Shapiro AD , Tyack PL , Solow AR . 2004 Call-type matching in vocal exchanges of free-ranging resident killer whales, Orcinus orca. Anim. Behav. **67**, 1099–1107. (10.1016/j.anbehav.2003.06.017)

[5] Johnson M , Aguilar de Soto N , Madsen P . 2009 Studying the behaviour and sensory ecology of marine mammals using acoustic recording tags: a review. Mar. Ecol. Prog. Ser. **395**, 55–73. (10.3354/meps08255)

[6] Johnson M , Madsen PT , Zimmer WMX , de Soto NA , Tyack PL . 2006 Foraging Blainville’s beaked whales (Mesoplodon densirostris) produce distinct click types matched to different phases of echolocation. J. Exp. Biol. **209**, 5038–5050. (10.1242/jeb.02596) 17142692

[7] Kragh IM , McHugh K , Wells RS , Sayigh LS , Janik VM , Tyack PL , Jensen FH . 2019 Signal-specific amplitude adjustment to noise in common bottlenose dolphins (Tursiops truncatus). J. Exp. Biol. **222**, 23. (10.1242/jeb.216606) 31704900

[8] Madsen PT , de Soto NA , Arranz P , Johnson M . 2013 Echolocation in Blainville’s beaked whales (Mesoplodon densirostris). J. Comp. Physiol. A Neuroethol. Sens. Neural. Behav. Physiol. **199**, 451–469. (10.1007/s00359-013-0824-8) 23636808

[9] Oliveira C , Wahlberg M , Johnson M , Miller PJO , Madsen PT . 2013 The function of male sperm whale slow clicks in a high latitude habitat: communication, echolocation, or prey debilitation? J. Acoust. Soc. Am. **133**, 3135–3144. (10.1121/1.4795798) 23654416

[10] Jensen FH , Perez JM , Johnson M , Soto NA , Madsen PT . 2011 Calling under pressure: short-finned pilot whales make social calls during deep foraging dives. Proc. Biol. Sci. **278**, 3017–3025. (10.1098/rspb.2010.2604) 21345867 PMC3158928

[11] Pérez JM , Jensen FH , Rojano‐Doñate L , Aguilar de Soto N . 2017 Different modes of acoustic communication in deep‐diving short‐finned pilot whales (Globicephala macrorhynchus). Marine. Mam. Sci. **33**, 59–79. (10.1111/mms.12344)

[12] O’Bryan LR , Abaid N , Nakayama S , Dey T , King AJ , Cowlishaw G , Rubenstein DI , Garnier S . 2019 Contact calls facilitate group contraction in free-ranging goats (Capra aegagrus hircus). Front Ecol. Evol. **7**, 73. (10.3389/fevo.2019.00073)

[13] Gero S , Whitehead H , Rendell L . 2016 Individual, unit and vocal clan level identity cues in sperm whale codas. R. Soc. Open Sci. **3**, 150372. (10.1098/rsos.150372) 26909165 PMC4736920

[14] Schulz TM , Whitehead H , Gero S , Rendell L . 2011 Individual vocal production in a sperm whale (Physeter macrocephalus) social unit. Marine. Mamm. Sci. **27**, 149–166. (10.1111/j.1748-7692.2010.00399.x)

[15] Oleson EM , Calambokidis J , Burgess WC , McDonald MA , LeDuc CA , Hildebrand JA . 2007 Behavioral context of call production by eastern North Pacific blue whales. Mar. Ecol. Prog. Ser. **330**, 269–284. (10.3354/meps330269)

[16] Parks SE , Searby A , Célérier A , Johnson MP , Nowacek DP , Tyack PL . 2011 Sound production behavior of individual North Atlantic right whales: implications for passive acoustic monitoring. Endang. Species Res. **15**, 63–76. (10.3354/esr00368)

[17] Goldbogen JA , Stimpert AK , DeRuiter SL , Calambokidis J , Friedlaender AS , Schorr GS , Moretti DJ , Tyack PL , Southall BL . 2014 Using accelerometers to determine the calling behavior of tagged baleen whales. J. Exp. Biol. **217**, 2449–2455. (10.1242/jeb.103259) 24803468

[18] Saddler MR , Bocconcelli A , Hickmott LS , Chiang G , Landea-Briones R , Bahamonde PA , Howes G , Segre PS , Sayigh LS . 2017 Characterizing Chilean blue whale vocalizations with DTAGs: a test of using tag accelerometers for caller identification. J. Exp. Biol. **220**, 4119–4129. (10.1242/jeb.151498) 28883086

[19] Stimpert AK , *et al* . 2015 Sound production and associated behavior of tagged fin whales (Balaenoptera physalus) in the Southern California Bight. Anim. Biotelemetry **3**, 1–12. (10.1186/s40317-015-0058-3)

[20] Dawbin WH . 1966 Whales, dolphins, and porpoises. In The seasonal migratory cycle of humpback whales (ed KS Norris ), Berkeley and Los Angeles: University of California Press. (10.1525/9780520321373)

[21] Dunlop RA , Noad MJ , Cato DH , Stokes D . 2007 The social vocalization repertoire of east Australian migrating humpback whales (Megaptera novaeangliae). J. Acoust. Soc. Am. **122**, 2893–2905. (10.1121/1.2783115) 18189579

[22] Stimpert AK , Au WWL , Parks SE , Hurst T , Wiley DN . 2011 Common humpback whale (Megaptera novaeangliae) sound types for passive acoustic monitoring. J. Acoust. Soc. Am. **129**, 476–482. (10.1121/1.3504708) 21303027

[23] Payne RS , McVay S . 1971 Songs of humpback whales. Science **173**, 585–597. (10.1126/science.173.3997.585) 17833100

[24] Stanistreet JE , Risch D , Van Parijs SM . 2013 Passive acoustic tracking of singing humpback whales (Megaptera novaeangliae) on a northwest Atlantic feeding ground. PLoS One **8**, e61263. (10.1371/journal.pone.0061263) 23593447 PMC3622601

[25] Rekdahl ML , Dunlop RA , Goldizen AW , Garland EC , Biassoni N , Miller P , Noad MJ . 2015 Non-song social call bouts of migrating humpback whales. J. Acoust. Soc. Am. **137**, 3042–3053. (10.1121/1.4921280) 26093396 PMC4474945

[26] Cusano DA , Indeck KL , Noad MJ , Dunlop RA . 2022 Humpback whale (Megaptera novaeangliae) social call production reflects both motivational state and arousal. Bioacoustics **31**, 17–40. (10.1080/09524622.2020.1858450)

[27] Demartsev V , Strandburg-Peshkin A , Ruffner M , Manser M . 2018 Vocal turn-taking in meerkat group calling sessions. Curr. Biol. **28**, 3661–3666. (10.1016/j.cub.2018.09.065) 30416063

[28] Takahashi DY , Narayanan DZ , Ghazanfar AA . 2013 Coupled oscillator dynamics of vocal turn-taking in monkeys. Curr. Biol. **23**, 2162–2168. (10.1016/j.cub.2013.09.005) 24139740

[29] Marques TA , Thomas L , Martin SW , Mellinger DK , Ward JA , Moretti DJ , Harris D , Tyack PL . 2013 Estimating animal population density using passive acoustics. Biol. Rev. Camb. Philos. Soc. **88**, 287–309. (10.1111/brv.12001) 23190144 PMC3743169

[30] Johnson MP , Tyack PL . 2003 A digital acoustic recording tag for measuring the response of wild marine mammals to sound. IEEE J. Oceanic. Eng. **28**, 3–12. (10.1109/JOE.2002.808212)

[31] Friedlaender A , Bocconcelli A , Wiley D , Cholewiak D , Ware C , Weinrich M , Thompson M . 2011 Underwater components of humpback whale bubble-net feeding behaviour. Behaviour **148**, 575–602. (10.1163/000579511X570893)

[32] Weinrich MT . 1991 Stable social associations among humpback whales (Megaptera novaeangliae) in the southern Gulf of Maine. Can. J. Zool. **69**, 3012–3019. (10.1139/z91-425)

[33] Weinrich MT , Schilling MR , Belt CR . 1992 Evidence for acquisition of a novel feeding behaviour: lobtail feeding in humpback whales, Megaptera novaeangliae. Animal Behaviour **44**, 1059–1072. (10.1016/S0003-3472(05)80318-5)

[34] Katona SK , Whitehead HP . 1981 Identifying humpback whales using their natural markings. Polar. Record. **20**, 439–444. (10.1017/S003224740000365X)

[35] Bérubé M , Palsbøll P . 1996 Identification of sex in cetaceans by multiplexing with three ZFX and ZFY specific primers. Mol. Ecol. **5**, 283–287. (10.1111/j.1365-294x.1996.tb00315.x) 8673273

[36] Palsbøll PJ , Vader A , Bakke I , El-Gewely MR . 1992 Determination of gender in cetaceans by the polymerase chain reaction. Can. J. Zool. **70**, 2166–2170. (10.1139/z92-292)

[37] Glockner DA . 1983 Determining the sex of humpback whales (*Megaptera novaeangliae*) in their natural environment. In Communication and behavior of whales (ed R Payne ), pp. 447–464, Boulder, CO: Westview Press.

[38] Chittleborough RG . 1959 Determination of age in the humpback whale, Megaptera nodosa (Bonnaterre). Mar. Freshwater Res. **10**, 125. (10.1071/MF9590125)

[39] Clapham P . 1992 Age attainment sexual maturity humpback whales, Megaptera novaeangliae. Can. J. Zool. **70**, 1470–1472. (10.1139/z92-202)

[40] Robbins, J. (2007). Structure and dynamics of the Gulf of Maine humpback whale population. http://research-repository.st-andrews.ac.uk/

[41] K. Lisa Yang Center for Conservation Bioacoustics at the Cornell Lab of Ornithology . 2023 Raven pro: interactive sound analysis software re (Version 2.0.0 Build 67 Beta). Ithaca, NY: The Cornell Lab of Ornithology. See https://ravensoundsoftware.com

[42] The MathWorks Inc . 2022 *MATLAB version: 9.7.0.1190202 (R2019B)*. Natick, Massachusetts: The MathWorks Inc. See https://www.mathworks.com

[43] Jensen FH . 2024 DTAGaudit_synch (Version 1.0.0). Computer software.

[44] R Core Team . 2021 R: A language and environment for statistical computing. Vienna, Austria: R Foundation for Statistical Computing. See https://www.R-project.org

[45] Sibly RM , Nott HMR , Fletcher DJ . 1990 Splitting behaviour into bouts. Anim. Behav. **39**, 63–69. (10.1016/S0003-3472(05)80726-2)

[46] Luque SP . 2007 Diving behaviour analysis in R. R News **7**, 8–14. https://cran.r-project.org/doc/Rnews

[47] Luque SP , Guinet C . 2007 A maximum likelihood approach for identifying dive bouts improves accuracy, precision and objectivity. Behaviour **144**, 1315–1332. (10.1163/156853907782418213)

[48] Gustison ML , Townsend SW . 2015 A survey of the context and structure of high- and low-amplitude calls in mammals. Anim. Behav. **105**, 281–288. (10.1016/j.anbehav.2015.04.021)

[49] Koren L , Geffen E . 2011 Individual identity is communicated through multiple pathways in male rock hyrax (Procavia capensis) songs. Behav. Ecol. Sociobiol. **65**, 675–684. (10.1007/s00265-010-1069-y)

[50] Cäsar C , Zuberbühler K , Young RJ , Byrne RW . 2013 Titi monkey call sequences vary with predator location and type. Biol. Lett. **9**, 20130535. (10.1098/rsbl.2013.0535) 24004492 PMC3971693

[51] Kershenbaum A , *et al* . 2016 Acoustic sequences in non-human animals: a tutorial review and prospectus. Biol. Rev. Camb. Philos. Soc. **91**, 13–52. (10.1111/brv.12160) 25428267 PMC4444413

[52] Fischer J , Hammerschmidt K , Todt D . 1995 Factors Affecting Acoustic Variation in Barbary‐macaque (Macaca sylvanus) Disturbance Calls. Ethology **101**, 51–66. (10.1111/j.1439-0310.1995.tb00345.x)

[53] Handel S , Todd SK , Zoidis AM . 2009 Rhythmic structure in humpback whale (Megaptera novaeangliae) songs: preliminary implications for song production and perception. J. Acoust. Soc. Am. **125**, EL225–EL230. (10.1121/1.3124712) 19507926

[54] Schneider JN , Mercado E . 2019 Characterizing the rhythm and tempo of sound production by singing whales. Bioacoustics **28**, 239–256. (10.1080/09524622.2018.1428827)

[55] Miller BS , *et al* . 2014 Blue whale vocalizations recorded around New Zealand: 1964-2013. J. Acoust. Soc. Am. **135**, 1616–1623. (10.1121/1.4863647) 24606296

[56] Gamba M , Torti V , Estienne V , Randrianarison RM , Valente D , Rovara P , Bonadonna G , Friard O , Giacoma C . 2016 The indris have got rhythm! timing and pitch variation of a primate song examined between sexes and age classes. Front. Neurosci. **10**, 249. (10.3389/fnins.2016.00249) 27378834 PMC4908265

[57] Zeh J , *et al* . 2024 Data from: Caller identification and characterization of individual Humpback whale acoustic behavior. Dryad Digital Repository. (10.5061/dryad.w9ghx3fw2) PMC10933536 38481982

